# Identification and validation of novel candidate genes with diagnostic value for sepsis via weighted gene co-expression network analysis

**DOI:** 10.3389/fmed.2026.1817161

**Published:** 2026-06-29

**Authors:** Xue Fu, Jian Yang, Qin Lv, Xiaotian Zhang, Sen Wang, Shangkun Cai, Meng Zhang

**Affiliations:** 1 Department of Emergency, Hebei Medical University Third Hospital, Shijiazhuang, China; 2 Department of Thoracic Surgery, Hebei Medical University Third Hospital, Shijiazhuang, China; 3 Faculty of Graduate Studies, Hebei Medical University, Shijiazhuang, China

**Keywords:** bioinformatics analysis, candidate genes, diagnostic value, immune cell infiltration, sepsis

## Abstract

**Background:**

Sepsis is a systemic inflammatory response caused by a variety of causes, which is characterized by high morbidity and mortality. Our work aimed to screen the candidate genes of sepsis and evaluate their diagnostic value using bioinformatics tools.

**Methods:**

Multiple GEO datasets were integrated. GSE9960 and GSE28750 were merged as the training set, with other datasets used as validation sets for immune infiltration, prognosis, ROC analysis, subtype analysis, and single-cell analysis. Sepsis-associated genes were identified via weighted gene co-expression network analysis (WGCNA) and protein–protein interaction (PPI) network analysis. Functional enrichment was explored using GSVA and GSEA. Diagnostic potential was evaluated by ROC curves. Correlation between gene expression and immune cells was analyzed by Pearson correlation, and gene expression was validated by qRT-PCR.

**Results:**

WGCNA identified 1,463 sepsis-associated genes, which were enriched in biosynthesis/metabolism and immune-related signaling pathways. Five hub genes (*CDK1*, *CCNB1*, *CCNA2*, *AURKB* and *BUB1*) were screened via PPI network, all highly expressed in sepsis. Higher *AURKB* or *BUB1* expression correlated with shorter overall survival. scRNA-seq revealed broad expression of these genes in pediatric immune cells (B cells, monocytes, T cells), but restricted to T cells in adult samples. A logistic regression model combining the five genes showed preliminary discriminative potential for distinguishing sepsis from healthy controls in the training set (AUC = 0.747) and an independent validation cohort (GSE65682, AUC = 0.799). In exploratory analyses, the model also demonstrated potential for differentiating septic shock from cardiogenic shock (AUC = 0.743) and from non-septic shock (AUC = 0.766). These genes exhibited significant correlations with immune cell infiltration in sepsis.

**Conclusion:**

In this study, we identified five sepsis-associated genes (*CDK1*, *CCNB1*, *CCNA2*, *AURKB*, *BUB1*). A logistic regression model based on these five genes demonstrated improved and consistent diagnostic performance for sepsis, and these genes also correlated with immune cell infiltration in sepsis. However, as the current results are correlational and do not establish a causal role, they should be interpreted as hypothesis-generating rather than conclusive, warranting further validation in independent prospective cohorts.

## 1 Introduction

Sepsis is a life-threatening complication following severe trauma, infection, or shock, often leading to multiple organ dysfunction syndrome (MODS) or septic shock (1). It is characterized by high morbidity and mortality, with clinical symptoms including high fever, shortness of breath, and rapid heartbeat (2). It has been estimated that approximately 48.9 million cases of sepsis were diagnosed and 11.0 million sepsis-related deaths occurred worldwide in 2017 (3). A study has found that the annual incidence of ICU-treated sepsis in adults is 290 per 100,000 individuals in Brazil, which produces approximately 420,000 cases of sepsis each year (4). Currently drug development in sepsis is mainly focused on regulating systemic inflammatory response, coagulopathy and immune dysfunction (5), such as cytokine antagonists (Afelimomab, CytoFab) (6) and recombinant Human soluble thrombosis regulators (7). However, the clinical treatment of sepsis includes antibiotics, antiviral drugs and vasoactive agents (8), while there is no specific therapeutic drug for clinical treatment of sepsis. Moreover, the diagnosis of sepsis is difficult because of the complex pathogenesis and multiple comorbidities (9). Therefore, identifying effective candidate genes and diagnostic methods is essential to improve cure rates and reduce mortality in sepsis.

Currently, the biomarkers of sepsis are mainly related to infection (procalcitonin, C-reactive protein, cytokines) (10, 11), inflammation activation and immune imbalance (monocyte chemoattractant P-1, programmed death receptor-1 and programmed death ligand-1) (12, 13), organ dysfunction (angiopoietin, matrix metalloproteinases) (14, 15) and so on. In addition, it has been demonstrated that the target genes exhibit diagnostic and prognostic value of sepsis. Wang et al. have shown that the expression levels of *UBE2D1* and *COX7C* are increased in diabetes-related sepsis compared to the normal group (16). Ding et al. (17) have found that the sepsis with *NEDD4L* and *SIAH2* high expression display worse prognosis, and the expression of *NEDD4L* and *SIAH2* are positively associated with M0 macrophages and negatively correlated with neutrophils. The expression level of *LPIN1* has been reported to be associated with immune cell infiltration in sepsis (18). Although the diagnosis of sepsis has made some progress, there is currently no effective method to diagnose sepsis accurately and quickly (19). Thus, it is necessary to study the molecular changes in the pathogenesis of sepsis and discover new effective biomarkers to support the early diagnosis and treatment of sepsis. Recent bioinformatics studies have demonstrated the value of integrated multi-omics approaches in identifying key genes and potential therapeutic targets for various diseases (20–23), supporting the rationale of the present study.

In this study, we analyzed sepsis samples from the Gene Expression Omnibus (GEO) database. We used weighted gene co-expression network analysis (WGCNA) and protein–protein interaction (PPI) network analysis, we identified hub genes associated with sepsis and evaluated their diagnostic potential via receiver operating characteristic (ROC) curves. We also investigated immune cell infiltration using CIBERSORT and examined correlations between hub gene expression and immune cells, aiming to provide new insights for the early diagnosis and prevention of sepsis.

## 2 Methods

### 2.1 Data sources

Multiple gene expression datasets were downloaded from the Gene Expression Omnibus (GEO) database.^1^ For training and immune infiltration analyses, four datasets were used: GSE9960 (54 sepsis, 16 healthy), GSE28750 (10 sepsis, 20 healthy), GSE134347 (156 sepsis, 83 healthy), and GSE13904 (52 sepsis, 18 healthy). To establish a training cohort, GSE9960 and GSE28750 were merged. Subsequently, batch effects between the combined datasets were corrected using the ComBat function from the sva package (version 3.40.0) with parameters mod = model.matrix(~1, data = pheno) (assuming no covariates) and par.prior = TRUE. The removeBatchEffect function from the limma package (version 3.48.0) was used only for PCA visualization to assess the effect of batch correction, and was not applied as a formal correction step in downstream analyses. For prognostic evaluation, GSE65682 (479 sepsis samples with 28-day survival information, and 42 healthy samples) was used independently.

To explore the expression patterns of hub genes across different shock types, GSE154918 (19 septic shock vs. 20 general sepsis), GSE131411 (28 septic shock vs. 34 cardiogenic shock), and GSE131761 (81 septic shock vs. 33 non-septic shock) were additionally analyzed. For single-cell RNA sequencing (scRNA-seq) analysis, the GSE279452 dataset was obtained, comprising three adult sepsis and three pediatric sepsis samples. A complete summary of all datasets, including sample sizes, platforms, and specific purposes, is provided in Table 1.

**Table 1:** Summary of datasets used in this study.

Dataset	Sample size (sepsis/healthy/other)	Platform	Purpose in this study
GSE9960	54 sepsis/16 healthy	GPL570	Training set (merged with GSE28750)
GSE28750	10 sepsis/20 healthy	GPL570	Training set (merged with GSE9960)
GSE134347	156 sepsis/83 healthy	GPL17586	Validation set for expression and immune infiltration
GSE13904	52 sepsis/18 healthy	GPL570	Validation set for immune infiltration
GSE65682	479 sepsis/42 healthy	GPL13667	Prognostic analysis (28-day survival) and ROC validation
GSE154918	19 septic shock/20 general sepsis	GPL20301	Septic shock vs. general sepsis expression analysis, ROC validation
GSE131411	28 septic shock/34 cardiogenic shock	GPL10999	Septic shock vs. cardiogenic shock expression analysis, ROC validation
GSE131761	81 septic shock/33 non-septic shock	GPL13497	Septic shock vs. non-septic shock expression analysis
GSE279452	3 adult sepsis, 3 pediatric sepsis	GPL24676	Single-cell RNA-seq analysis

Probe IDs were converted to gene symbols using the platform-provided annotation files. For multiple probes mapping to the same gene, the probe with the highest median expression level was retained. For probes without annotation information, re-annotation was performed based on alignment positions to the reference genome (e.g., hg38).

### 2.2 Weighted gene co-expression network analysis (WGCNA)

Before constructing the co-expression network, sample outliers were detected based on a hierarchical clustering dendrogram using the hclust function. Outlier samples were removed using the cutreeStatic function with a height threshold of >150. Based on the expression values of genes, the top 25% of genes (ranked by median absolute deviation (MAD) to retain highly dynamic genes and enhance network robustness, a threshold reference following common WGCNA practices) were screened by variance analysis for WGCNA using the “WGCNA” package (24) in R language (version 4.2.1, the same below). Next, Pearson correlation coefficients were calculated for each gene, and an appropriate soft threshold β was selected. The one-step method was applied to build the gene network, and the adjacency matrix was transformed into a topological overlap matrix (TOM). Hierarchical clustering was applied to produce a hierarchical clustering tree. Initial modules were identified using the dynamic tree cut algorithm (cutreeDynamic) with parameters set as deepSplit = 4 and minClusterSize = 30. Subsequently, modules were merged based on the correlation of module eigengenes: when the correlation coefficient between two module eigengenes was > 0.75, they were merged. Genes with intramodular connectivity (kME) > 0.8 were defined as significantly correlated genes. The significance of genes and modules was calculated to measure the relationship between genes and clinical information, and the significant association between modules and traits was analyzed to obtain gene modules.

### 2.3 Functional analysis

Gene set variation analysis (GSVA) and Gene set enrichment analysis (GSEA) between the sepsis and healthy groups were performed by “GSVA” (25) and “ClusterProfiler” (26) packages in R language, respectively. The gene set used for GSVA was c2.cp.kegg.v2022.1.hs. Symbols, downloaded from the Molecular Signatures Database.^2^ For GSEA, 1000 gene set permutations were performed by default, and the false discovery rate (FDR) was used to control the type I error rate, with a significance threshold of q < 0.25. After GSVA, differential pathway comparisons were performed using the eBayes test in the Limma package with Benjamini-Hochberg (BH) correction. A adjusted *p* < 0.05 was considered significant.

### 2.4 Protein–protein interaction (PPI) network analysis

The STRING (https://string-db.org/, version 11.0) database (27) was employed to analyze the functional links between proteins. The Cytoscape (version 3.7.2) (28) was applied to visualize the PPI network. Moreover, based on the maximum neighborhood component (MNC) algorithm, the CytoHubba plugin in Cytoscape was used to further screen the hub genes in PPI network.

### 2.5 Single-cell RNA sequencing (scRNA-seq) data acquisition and preprocessing

ScRNA-seq data comprising sepsis adult (GSM8571091, GSM8571092, GSM8571093) and sepsis pediatric samples (GSM8571082, GSM8571083, and GSM8571084) were obtained from the publicly available dataset GSE279452. Data processing was performed using the Seurat package (v4.0) in R (29). Low-quality cells were filtered out based on the following criteria: nFeature_RNA > 200, nFeature_RNA < 5,000, and percent.mt < 10%. Following data normalization using the “NormalizeData” function, principal component analysis (PCA) was performed with “RunPCA.” Major cell subpopulations were then identified through unsupervised clustering via the “FindClusters” function in Seurat and visualized using Uniform Manifold Approximation and Projection (UMAP). Automated cell type annotation was subsequently carried out with the SingleR package. Cells were used as the observation unit for visualization only. No formal statistical comparisons (e.g., *t*-test or Wilcoxon) were performed between adult and pediatric groups at the single-cell level, as cells from the same donor are not independent observations. The expression patterns are presented for descriptive purposes only.

### 2.6 Clinical sample collection

Whole blood samples from 20 sepsis and 20 healthy controls were collected at the Third Hospital of Hebei Medical University from September 18, 2024 to October 4, 2024. Sepsis was diagnosed according to the Sepsis-3 criteria, defined as confirmed or suspected infection with an acute increase of ≥ 2 points in the SOFA score. Each participant signed the appropriate informed consent form. All research activities were approved by the Ethics Committee of the Third Hospital of Hebei Medical University (KS2024-236-1), and informed consent was obtained from all participants. The clinical characteristics of the patients are presented in Table 2.

**Table 2:** Clinical information of sepsis and healthy individuals.

Clinical characteristics	Sepsis (*N* = 20)	Healthy (*N* = 20)
Age (years)	60.55 ± 14.92	33.05 ± 9.74
Female, *n* (%)	4 (20%)	12 (60%)
Body mass index	23.96 ± 3.01	25.30 ± 4.35
White cell count on admission (x10^9^)	11.49 ± 4.62	
SOFA score on admission, median (IQR)	8.70 ± 4.33	
APACHE II score on admission	19.50 ± 5.05	
CRP on admission (mg/L)	52.20 ± 68.99	
Pulmonary infection site, n (%)	20 (100%)	

SOFA, Sequential organ failure assessment; APACHE II, Acute physiology and chronic health evaluation II; CRP, C-reactive Protein. CRP data were available for 16 of 20 patients.

### 2.7 RT-qPCR assay

Total RNA was extracted from whole blood samples using the high-efficiency blood total RNA extraction kit RNAprep pure (DP443, Tiangen Biotechnology Co., Ltd., Beijing, China). RNA purity and concentration were assessed by measuring the OD260/280 ratio using a spectrophotometer. Only samples with an OD260/280 ratio between 1.8 and 2.0 were used for subsequent analyses. Melting curve analysis was performed after each PCR run to confirm specific amplification. Evo M-MLV reverse transcription master mix kit (AG11728, Accurate Biology, Changsha, China) was used for the reverse transcription process. Subsequent qRT-PCR detection was performed on a real-time quantitative fluorescence PCR instrument (SLAN-96S, Shanghai Hongshi Medical Technology Co., Ltd., Shanghai, China) using SuperStar Universal SYBR Master Mix (CW3360M, Jiangsu Kaowen Biotechnology Co., Ltd., Jiangsu, China). Detailed information on the primers used can be found in Table 3. The cycling protocol consisted of an initial predenaturation phase at 95 °C for 30 s, followed by 40 cycles alternating between 95 °C for 10 s and 60 °C for 30 s. GAPDH functions as an internal control. The 2^-ΔCT^ method was used to evaluate relative mRNA expression levels.

**Table 3:** Primer sequences for RT-PCR

Genes	Forward Primer (5′-3′)	Reverse Primer (5′-3′)
CDK1	ACTACAGGTCAAGTGGTAGCC	TCCTGCATAAGCACATCCTGA
CCNB1	GCACTTCCTTCGGAGAGCAT	TGTTCTTGACAGTCCATTCACCA
CCNA2	CCGTTCCTCCTTGGAAAGCA	CAGGGCATCTTCACGCTCTACTT
AURKB	TCTTAACGCGGCACTTCACA	GTGGGCCTGGATTTCGATCT
BUB1	ACCCCGGAAAATGTCCTTCAAT	CAGAGGGGATGACAGGGTTC
GAPDH	GAAGGTGAAGGTCGGAGTC	GAAGATGGTGATGGGATTTC

### 2.8 Immune cell infiltration

The relative proportions of 22 human immune cells were calculated by CIBERSORT software (30). CIBERSORT outputs a *p*-value for each sample based on Monte Carlo sampling to assess the reliability of deconvolution. Only samples with *p* < 0.05 were included for subsequent group comparisons. In our analysis, all samples met this criterion and were retained. According to the gene expression matrix, CIBERSORT describes the composition of immune infiltrating cells using 547 preset barcode genes in a deconvolution algorithm. The sum of all estimated immune cell type proportions in each sample equals 1. Differences in immune cell abundances between groups were compared using the Wilcoxon test with BH correction (adjusted *p* < 0.05).

### 2.9 Statistical analysis

The differences in immune cell infiltration between the groups were compared by Wilcoxon rank sum test. Pearson correlation analysis was performed using the R language “cor” function. *p* < 0.05 were considered statistically significant. Multicollinearity in the logistic regression model was assessed using variance inflation factors (VIFs), with VIF < 5 indicating no significant collinearity.

## 3 Results

### 3.1 Identification of sepsis-associated genes

**Figure 1:**
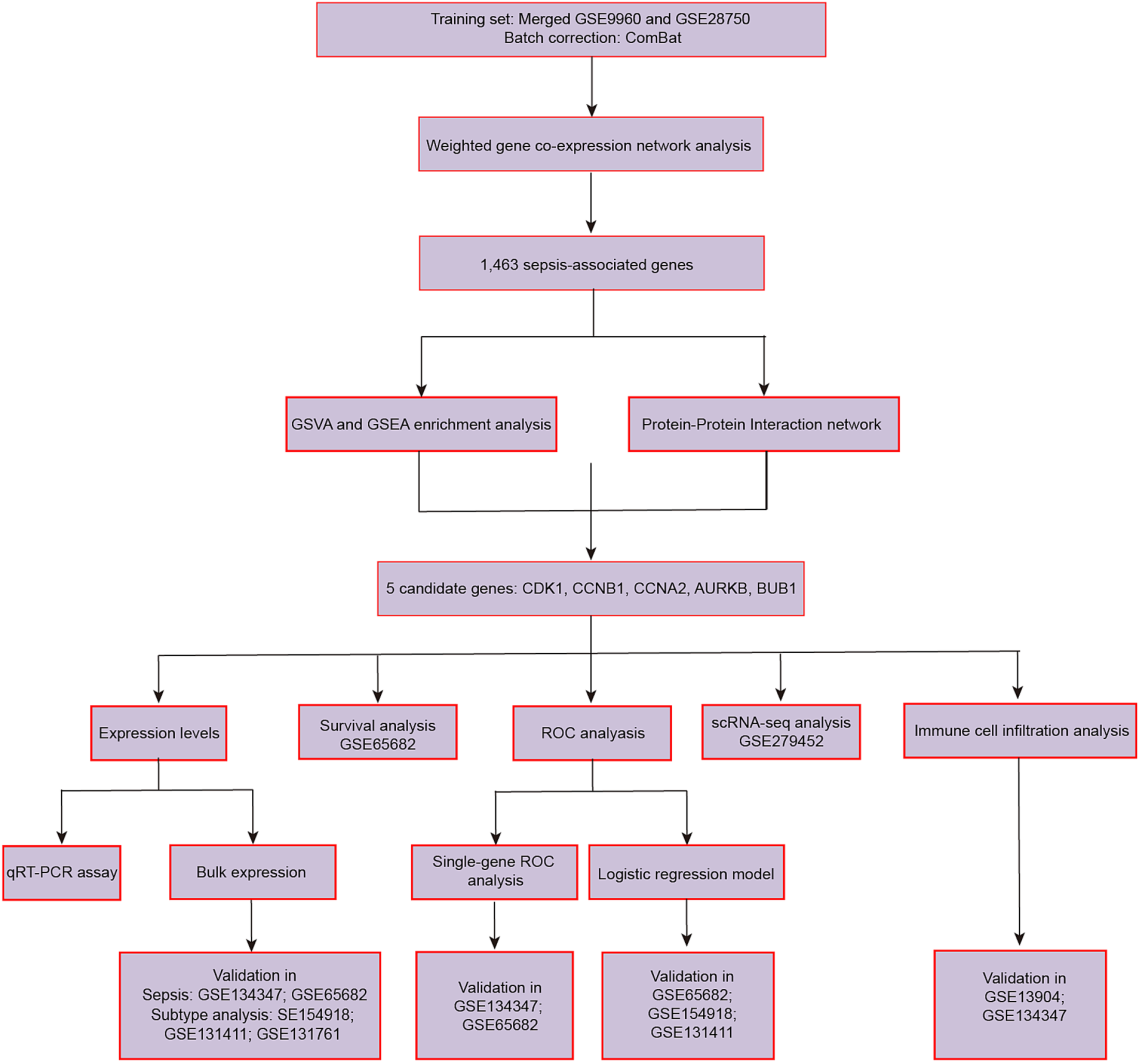
The flow chart of this study.

**Figure 2:**
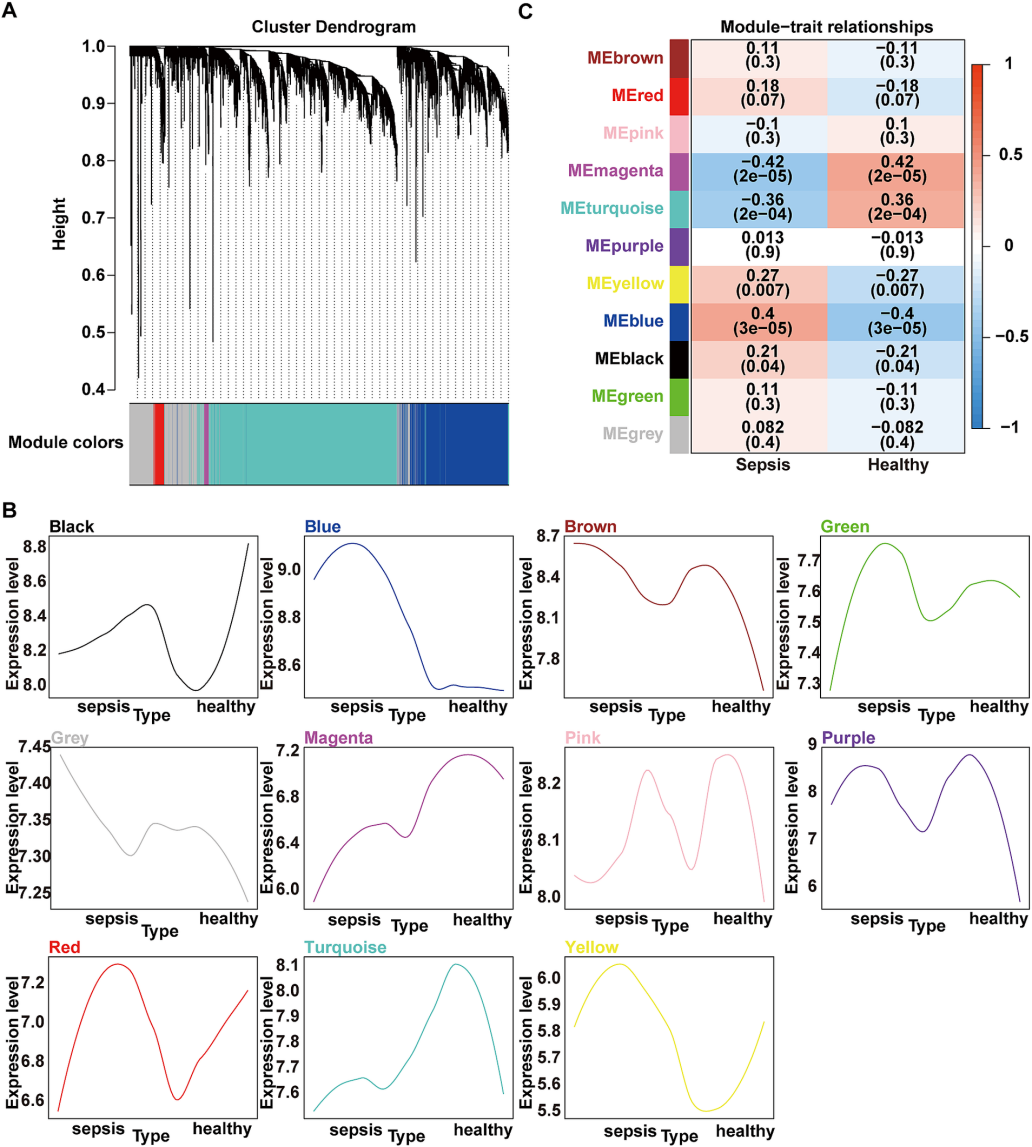
The result of weighted gene co-expression network analysis (WGCNA). **(A)** The clustering result of gene module, the top half is a hierarchical clustering dendrogram of genes, the bottom half is gene modules, module colors represent the color of each module, and the gray module is a collection of genes that cannot be clustered to other modules. **(B)** The expression trend of 11 module gene. **(C)** The correlation of gene models and sepsis and healthy samples. Significance markers represent unadjusted *p* values.

The flow chart of this study is shown in Figure 1. Firstly, we merged the GSE9960 and GSE28750 datasets as a training set, and the batch effects were removed (Supplementary Figure S1). To identify highly correlated genes associated with the onset of sepsis, WGCNA were performed using samples from the training cohort. *β* = 16 was chosen as the optimal soft threshold for constructing the scale-free network (Supplementary Figure S2). Eleven gene modules were obtained by the WGCNA analysis (Figure 2A). The expression trends of the genes in each module were displayed in Figure 2B. Three gene modules (yellow, black, and blue) had significant positive correlation with sepsis among these 11 gene modules (Figure 2C). Finally, a total of 1,463 genes found within the yellow, black, blue modules were considered as sepsis-associated genes for additional investigation (Supplementary Table S1).

### 3.2 Functional enrichment analysis of sepsis-associated genes

**Figure 3:**
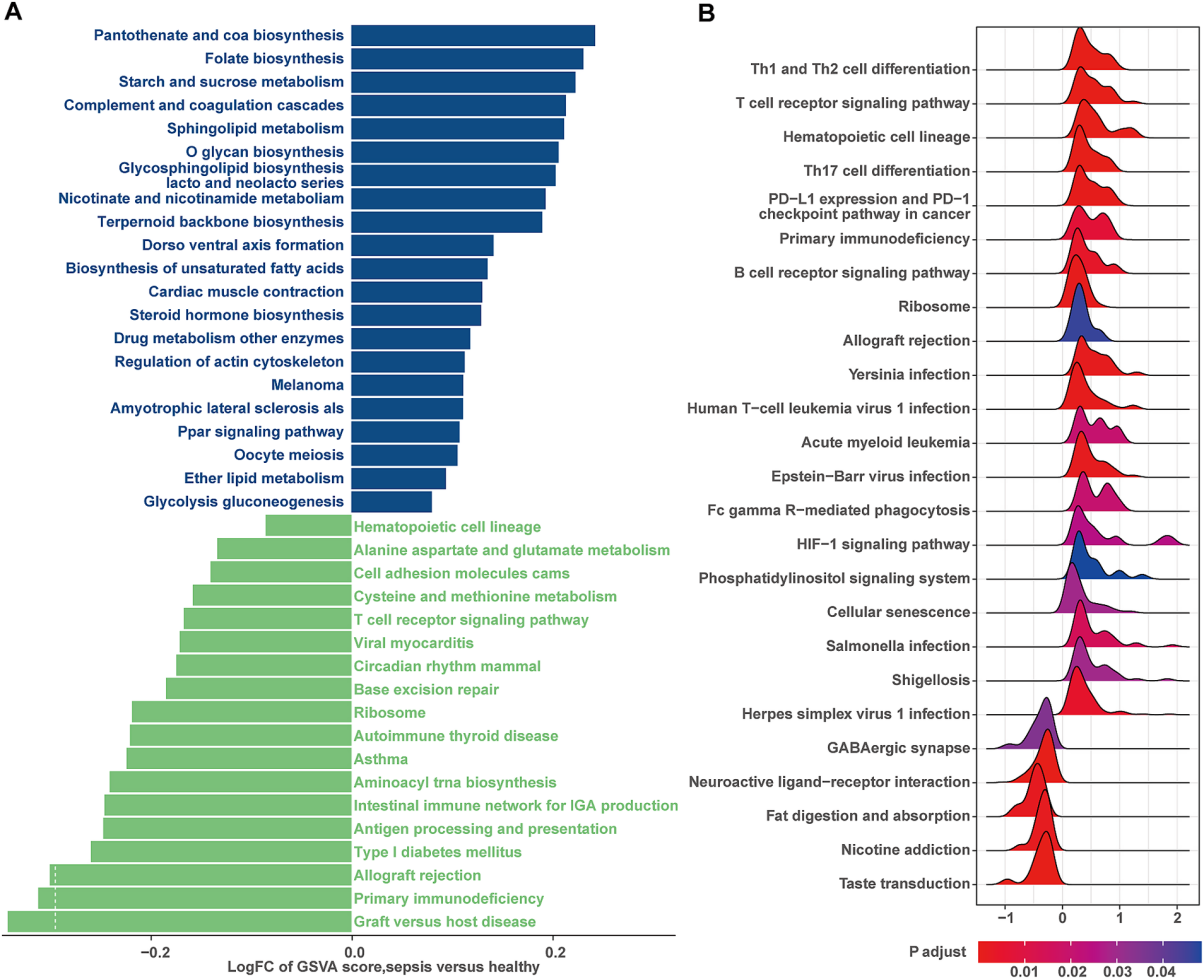
Functional enrichment analysis. **(A,B)**. The enriched pathways of 1,463 sepsis-associated genes using gene set variation analysis (GSVA) **(A)** and gene set enrichment analysis (GSEA) **(B)**. Significance markers represent adjusted *p* values (Benjamini-Hochberg correction).

To explore the functions of the 1,463 sepsis-associated genes, GSVA and GSEA were performed. GSVA revealed significant enrichment in biosynthesis and metabolism pathways, including pantothenate and CoA biosynthesis, folate biosynthesis, hematopoietic cell lineage, and alanine, aspartate, and glutamate metabolism (Figure 3A, Supplementary Table S2, *p* < 0.05). GSEA showed enrichment in immune-related signaling pathways, such as Th1 and Th2 cell differentiation, T cell receptor signaling, Th17 cell differentiation, and the HIF-1 signaling pathway (Figure 3B, Supplementary Table S2, *p* < 0.05).

### 3.3 Identification of candidate sepsis-associated genes

**Figure 4:**
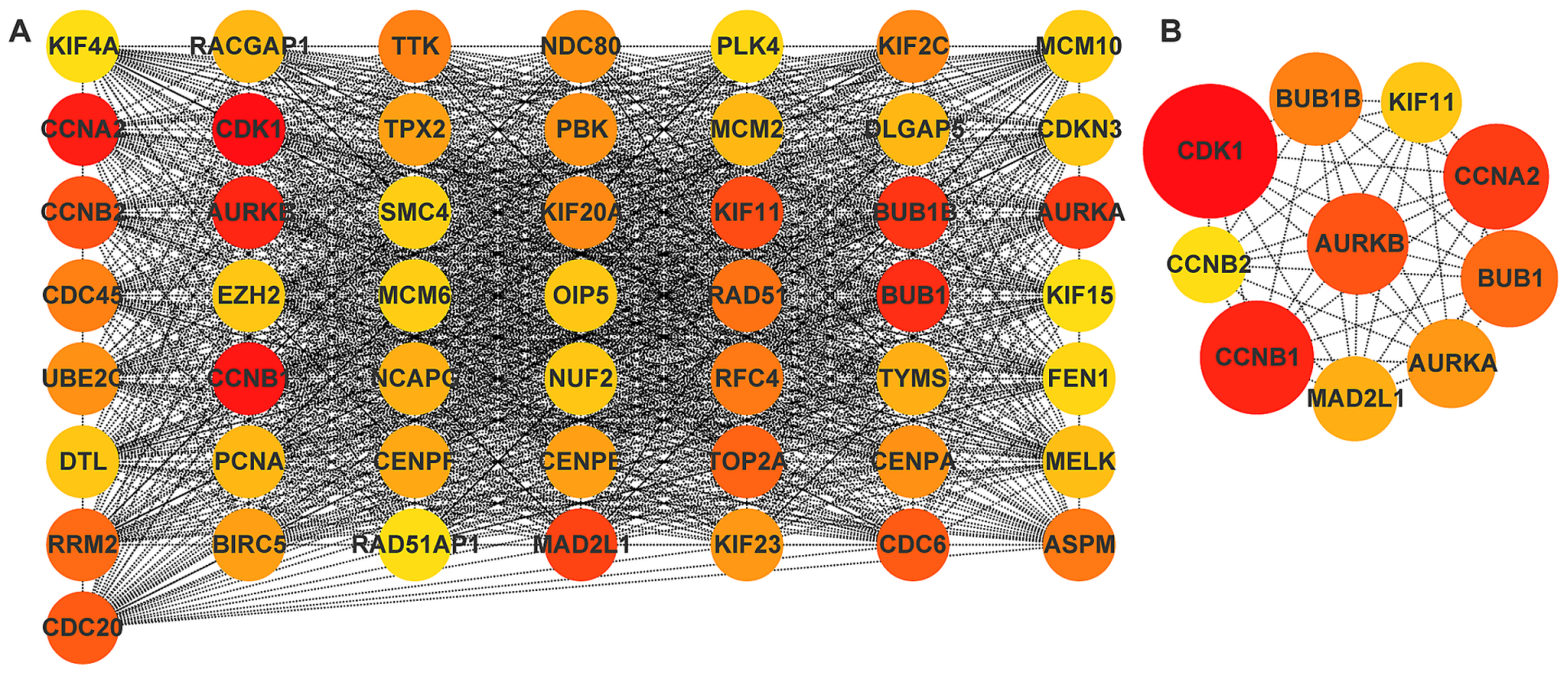
Construction of protein–protein interaction (PPI) network and identification of candidate genes associated with sepsis. **(A)** A PPI network of the top 50 hub genes. **(B)** Reciprocal PPI network of top 10 genes.

To further identify the hub genes among the 1,463 sepsis-associated genes, a PPI interaction network was constructed, and the interaction pairs were screened according to minimum required interaction score > 0.4. As shown in Supplementary Figure S3, the PPI network contained 1,335 nodes and 14,956 edges. A PPI network of the top 50 hub genes was shown in Figure 4A. Next, we analyzed the topological structure of the whole PPI network and scored the importance for each node in the network, and screened top 10 genes according to the score from largest to smallest (Figure 4B, Supplementary Table S3). The top 5 genes (*CDK1*, *CCNB1*, *CCNA2*, *AURKB*, and *BUB1*) with the highest significance of nodes were selected as hub candidate genes associated with sepsis. Notably, all five genes are canonical cell-cycle or mitosis-related genes, and they also ranked as the top five nodes in the PPI network based on degree connectivity (Supplementary Table S3).

### 3.4 CDK1, CCNB1, CCNA2, AURKB, BUB1 were up-regulated in sepsis

**Figure 5:**
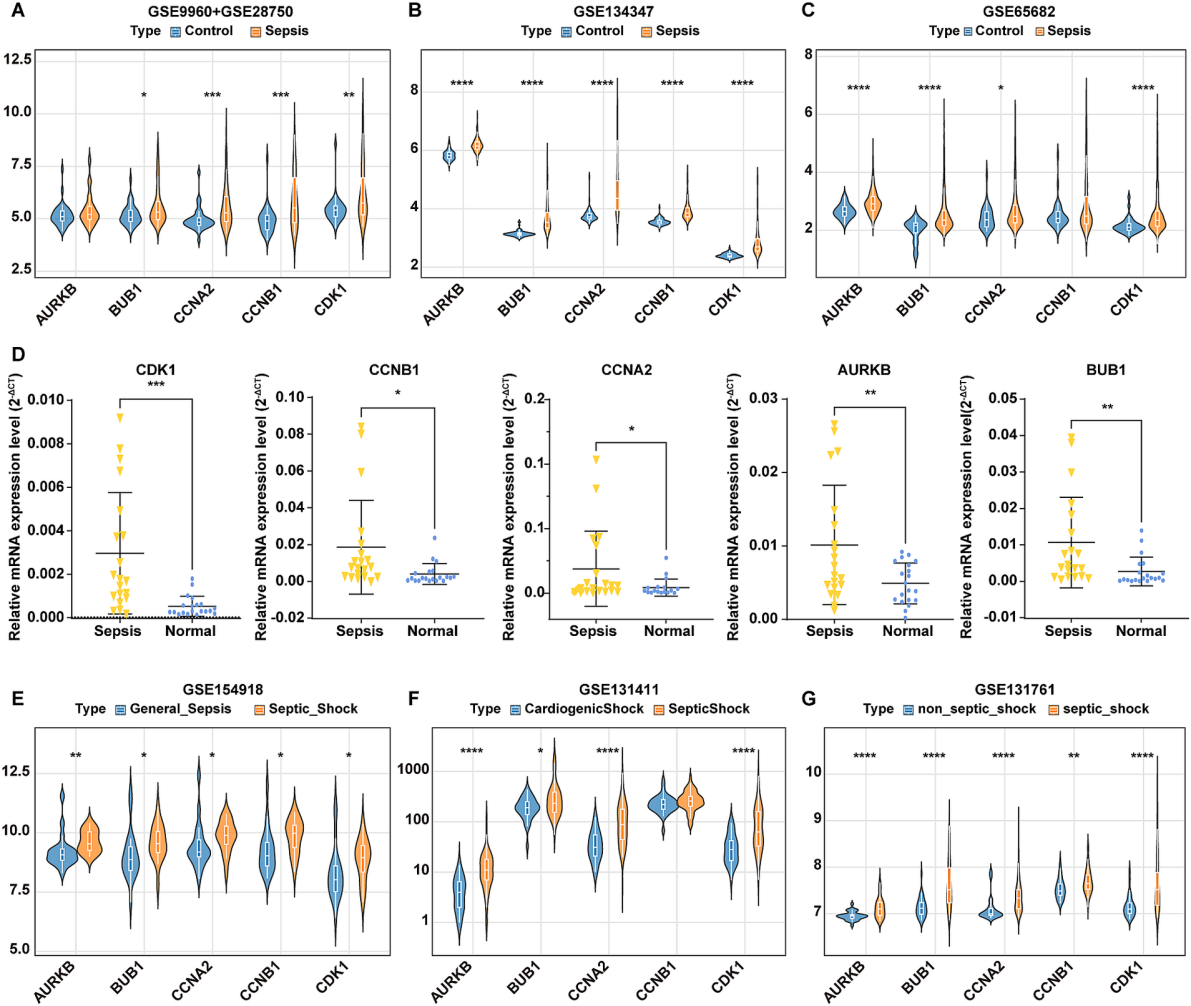
Expression levels of *CDK1*, *CCNB1*, *CCNA2*, *AURKB*, and *BUB1* in sepsis. **(A–C)** The expression of *CDK1*, *CCNB1*, *CCNA2*, *AURKB*, and *BUB1* in the training set **(A)**, GSE134347 **(B)**, and GSE65682 **(C)** validation sets. **(D)** Validation of mRNA expression in whole blood samples from sepsis patients (*N* = 20) and healthy controls (*N* = 20) by qRT-PCR. **(E)** The expression of *CDK1, CCNB1, CCNA2, AURKB, BUB1* in the septic shock and general sepsis samples in the GSE154918 dataset. **(F,G)** The expression of *CDK1*, *CCNB1*, *CCNA2*, *AURKB*, and *BUB1* in the septic shock and cardiogenic shock or non-septic shock samples. * means *p* < 0.05, ** means *p* < 0.01, *** means *p* < 0.001, **** means *p* < 0.0001, ns, no significance. Significance markers represent unadjusted p values.

To assess the expression patterns of the five candidate genes in sepsis, we analyzed their transcriptional levels across the training set (GSE9960 + GSE28750) and two independent validation cohorts (GSE134347 and GSE65682). In the training set, *BUB1, CCNA2*, *CCNB1*, and *CDK1* were significantly upregulated in sepsis samples compared with controls (Figure 5A). Consistent with this finding, the GSE134347 dataset revealed elevated expression of *AURKB*, *BUB1*, *CCNA2*, *CCNB1*, and *CDK1* in the sepsis group (Figure 5B). Similar trends were observed in GSE65682, where *AURKB*, *BUB1*, *CCNA2*, and *CDK1* showed significantly higher expression in sepsis samples (Figure 5C). To further verify this result, we analyzed the expression of these five genes in the blood samples of patients with sepsis and found that the mRNA expression levels of *CDK1, CCNB1, CCNA2, AURKB, BUB1* were increased in the blood samples of patients with sepsis (Figure 5D).

Furthermore, we explored the correlation of *CDK1*, *CCNB1*, *CCNA2*, *AURKB*, and *BUB1* with the severity of sepsis. In the GSE154918 dataset, *AURKB*, *BUB1*, *CCNA2*, *CCNB1*, and *CDK1* were significantly highly expressed in Septic Shock samples compared to general sepsis samples (Figure 5E). Moreover, we analyzed the role of *CDK1*, *CCNB1*, *CCNA2*, *AURKB*, and *BUB1* in distinguishing septic shock from non-septic shock. In the GSE131411 dataset, *AURKB*, *BUB1*, *CCNA2*, and *CDK1* were observably upregulated in septic shock samples compared to cardiogenic shock samples (Figure 5F). In the GSE131761 dataset, *AURKB*, *BUB1*, *CCNA2*, *CCNB1*, and *CDK1* were highly expressed in septic shock group compared to non- septic shock group (Figure 5G). These findings suggest that *CDK1*, *CCNB1*, *CCNA2*, *AURKB*, and *BUB1* may serve as potential candidate biomarkers for differentiating septic shock from other types of shock, though further validation is needed.

### 3.5 Diagnostic performance of single genes versus a multivariable logistic regression model, and prognostic relevance of individual genes

**Figure 6:**
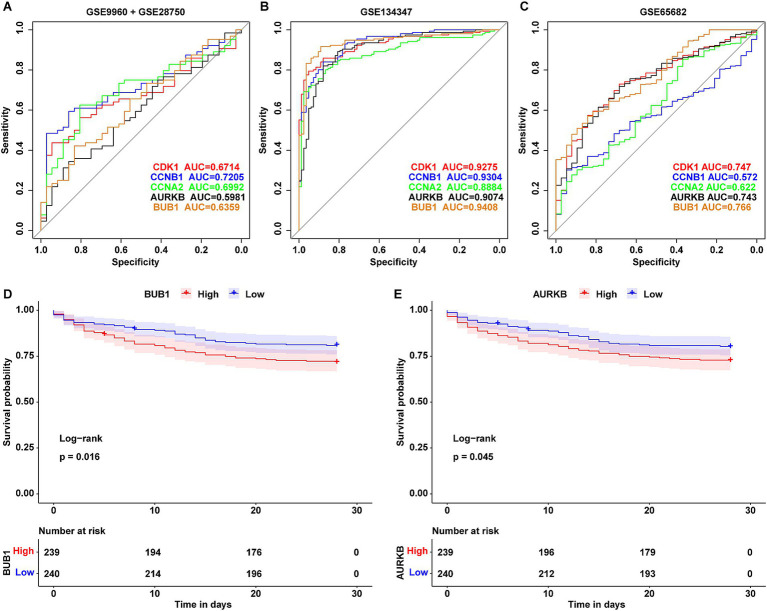
Diagnostic and prognostic value of the five candidate genes in sepsis. **(A–C)** The area under the curve (AUC) values of *CDK1*, *CCNB1*, *CCNA2*, *AURKB*, and *BUB1* in the training set **(A)** and GSE134347 **(B)**, GSE65682 **(C)** validation sets. **(D)** Kaplan–Meier (KM) curve of 28-day overall survival for sepsis patients stratified by *BUB1* expression (high vs. low). **(E)** KM curve of 28-day overall survival for sepsis patients stratified by *AURKB* expression (high vs. low). Significance markers represent unadjusted *p* values.

To evaluate the diagnostic potential of the five candidate genes for sepsis, ROC curves were constructed in the training set and two independent validation datasets (GSE134347 and GSE65682). In the training set, the area under the curve (AUC) values for *CDK1*, *CCNB1*, *CCNA2*, *AURKB*, and *BUB1* were 0.671, 0.720, 0.699, 0.598, and 0.636, respectively (Figure 6A). In the GSE134347 dataset, each gene achieved an AUC exceeding 0.88 (Figure 6B). In the GSE65682 cohort, the AUC values were 0.747 (*CDK1*), 0.572 (*CCNB1*), 0.622 (*CCNA2*), 0.743 (*AURKB*), and 0.766 (*BUB1*), respectively (Figure 6C).

Given this inconsistency, we constructed a multivariable logistic regression model incorporating the five candidate genes. First, we assessed the linearity assumption using component-plus-residual plots. As shown in Supplementary Figure S4A, the smooth fitted lines for each gene showed no obvious nonlinear trends, confirming that the linearity assumption was acceptable. The model was constructed using the training set and validated in multiple independent cohorts (GSE65682, GSE154918, and GSE131411). The model was constructed using the training set (GSE9960 + GSE28750) with the following formula: y = 4.0705 × *BUB1* + 2.3446 × *AURKB*+0.1343 × *CCNA2*-1.4276 × *CCNB1* + 2.3703 × *CDK1* − 14.5139. Model calibration was assessed using a calibration curve (Supplementary Figure S4B), which showed good agreement between predicted probabilities and observed outcomes. The regression coefficients, 95% confidence intervals, standard errors, z-values, and *p*-values for each variable are summarized in Table 4. The optimal cutoff value was 0.9718, with a corresponding sensitivity of 0.6414 and specificity of 0.8947 in the training set. To investigate the negative regression coefficient observed for *CCNB1* in the multivariable model, we calculated variance inflation factors (VIFs) for all five predictors. As shown in Table 4, the VIF for CCNB1 was 1.8, and VIFs for the remaining four genes ranged from 1.0 to 1.5. All VIFs were well below conventional thresholds (5 or 10), indicating no significant multicollinearity. Thus, the negative coefficient for CCNB1 is not a spurious result driven by collinearity. Rather, it represents the independent contribution of CCNB1 after controlling for the other four genes. This apparent discrepancy—a gene upregulated in sepsis but with a negative coefficient in the multivariable model—reflects the nature of multivariable logistic regression, where the model assigns positive and negative weights to different predictors to optimize the classification boundary.

**Table 4:** Multivariable logistic regression model coefficients, variance inflation factors (VIFs), and diagnostic performance.

Variable	Estimate	95%CI	Std_Error	Z value	*P* value	Vif	Threshold	Sensitivity	Specificity
(Intercept)	−14.5139	−20.7044572 to −9.0513443	2.9697	−4.887	1.02E-06	/	0.9718	0.6414	0.8947
CDK1	2.3703	0.5944076 to 4.3027963	0.9484	2.499	0.01245	1.3469			
CCNB1	−1.4276	−2.3531611 to −0.4506457	0.4817	−2.963	0.00304	1.8308			
CCNA2	0.1343	−1.3388872 to 1.6125367	0.751	0.179	0.045808	1.5429			
AURKB	2.3446	0.9984154 to 3.8192274	0.7177	3.267	0.00109	1.1277			
BUB1	4.0705	2.6678579 to 5.7182478	0.7741	5.258	1.45E-07	1.0422			

VIF, Variance inflation factors; VIFs for all five predictors were below 5, indicating no significant multicollinearity.

The model appeared to have some discriminatory potential for sepsis versus healthy controls in the training set (AUC = 0.747) and the GSE65682 validation cohort (AUC = 0.799) (Supplementary Figures S4C,D). In separate exploratory analyses using the GSE154918 and GSE131411 datasets, the model achieved AUCs of 0.766 and 0.743, respectively (Supplementary Figures S4E,F), suggesting that the five-gene signature may potentially be useful in differentiating septic shock from other shock types (cardiogenic shock and non-septic shock, respectively).

In addition to diagnostic performance, we also evaluated the prognostic relevance of these genes. Sepsis patients in the GSE65682 dataset were stratified into high- and low-expression groups based on the median expression level of each candidate gene. Kaplan–Meier survival analysis over 28 days revealed that patients with high expression of *BUB1* or *AURKB* exhibited significantly poorer survival compared to those with low expression (Figures 6D,E).

### 3.6 Expression patterns of hub genes in single-cell analysis reveal broader cell-type distribution in pediatric samples

**Figure 7:**
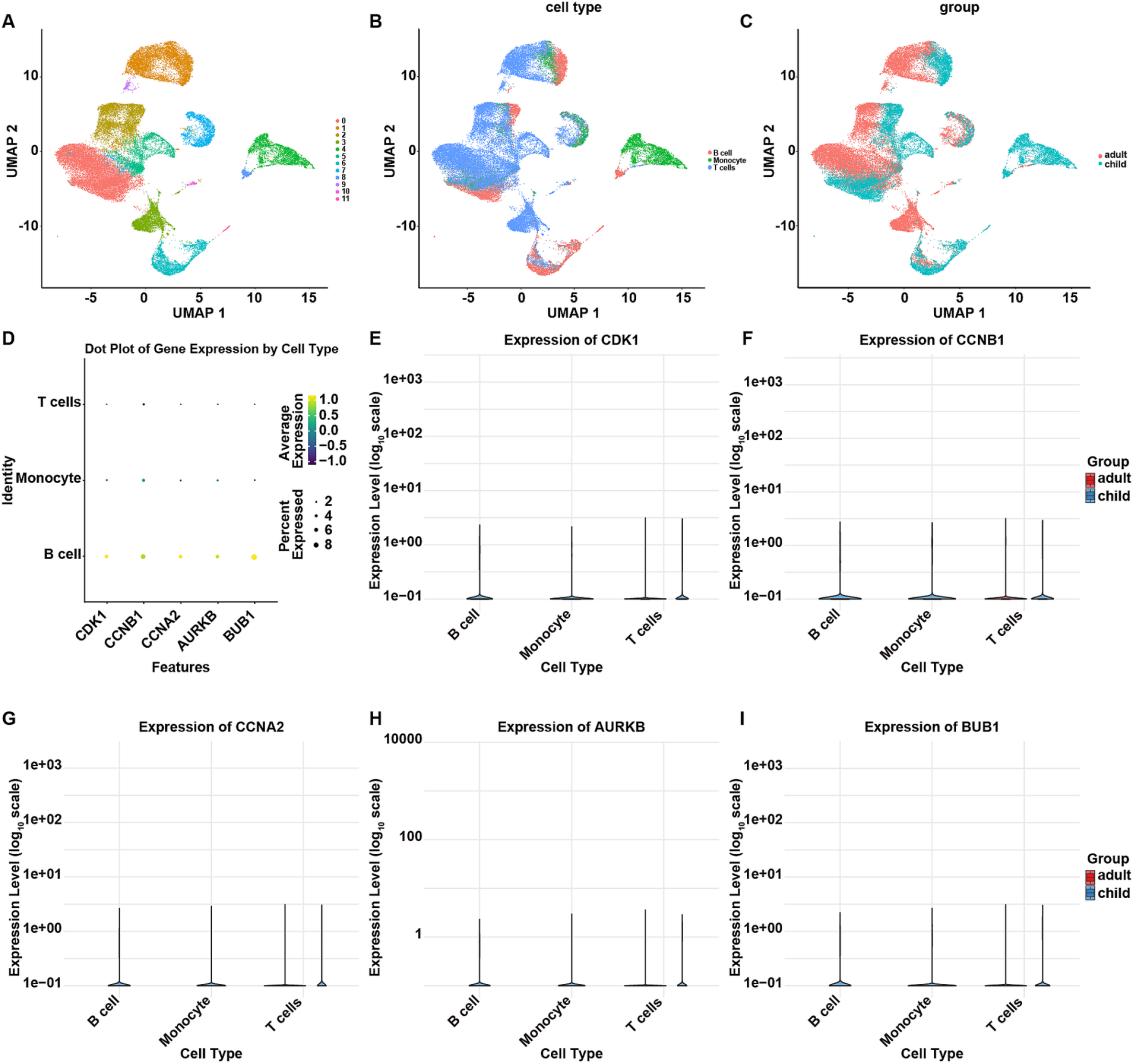
Expression patterns of candidate genes in single-cell analysis reveal broader cell-type distribution in pediatric samples. **(A)** UMAP plot showing 12 distinct clusters. **(B)** UMAP plot showing the distributions of 3 distinct cell types from all sepsis samples. **(C)** UMAP plot displays the distribution of cells derived from adult (*n* = 3) and pediatric (*n* = 3) sepsis patients. **(D)** Cell type-specific expression patterns of candidate genes in sepsis. **(E–I)** The expression of *CDK1*, *CCNB1*, *CCNA2*, *AURKB*, and *BUB1* in different cell types in the adult and pediatric sepsis samples. **** means *p* < 0.0001. Significance markers represent unadjusted p values and are shown for auxiliary purposes only; no formal statistical comparisons were performed at the single-cell level. The expression patterns are presented for descriptive purposes only.

To investigate the expression patterns of candidate genes (*CDK1, CCNB1*, *CCNA2*, *AURKB*, and *BUB1*) across different cell types in sepsis, we analyzed scRNA-seq data from the GSE279452 dataset, which includes three adult sepsis and three pediatric sepsis samples. After quality control and normalization, a total of 43,576 cells were clustered into 12 distinct clusters (Figure 7A). Based on established marker genes, the cells were annotated into three major cell types: B cells, monocytes, and T cells (Figures 7B,C). As shown in Figure 7D, *CDK1*, *CCNB1*, *CCNA2*, *AURKB*, and *BUB1* were predominantly expressed in B cells, followed by monocytes and T cells. We further compared gene expression between adult and pediatric samples across different cell types. As illustrated in Figures 7E–I, in pediatric samples, all five genes were broadly expressed in B cells, monocytes, and T cells. In contrast, in adult samples, expression of these genes was predominantly restricted to T cells and was largely absent in B cells and monocytes. No statistical comparisons between adult and pediatric samples were performed at the single-cell level; the expression patterns are presented for descriptive purposes only. Given the limited sample size (three adults, three children) and absence of healthy controls, these findings should be interpreted as exploratory.

### 3.7 CDK1, CCNB1, CCNA2, AURKB, and BUB1 were correlated with immune cell infiltration in sepsis

**Figure 8:**
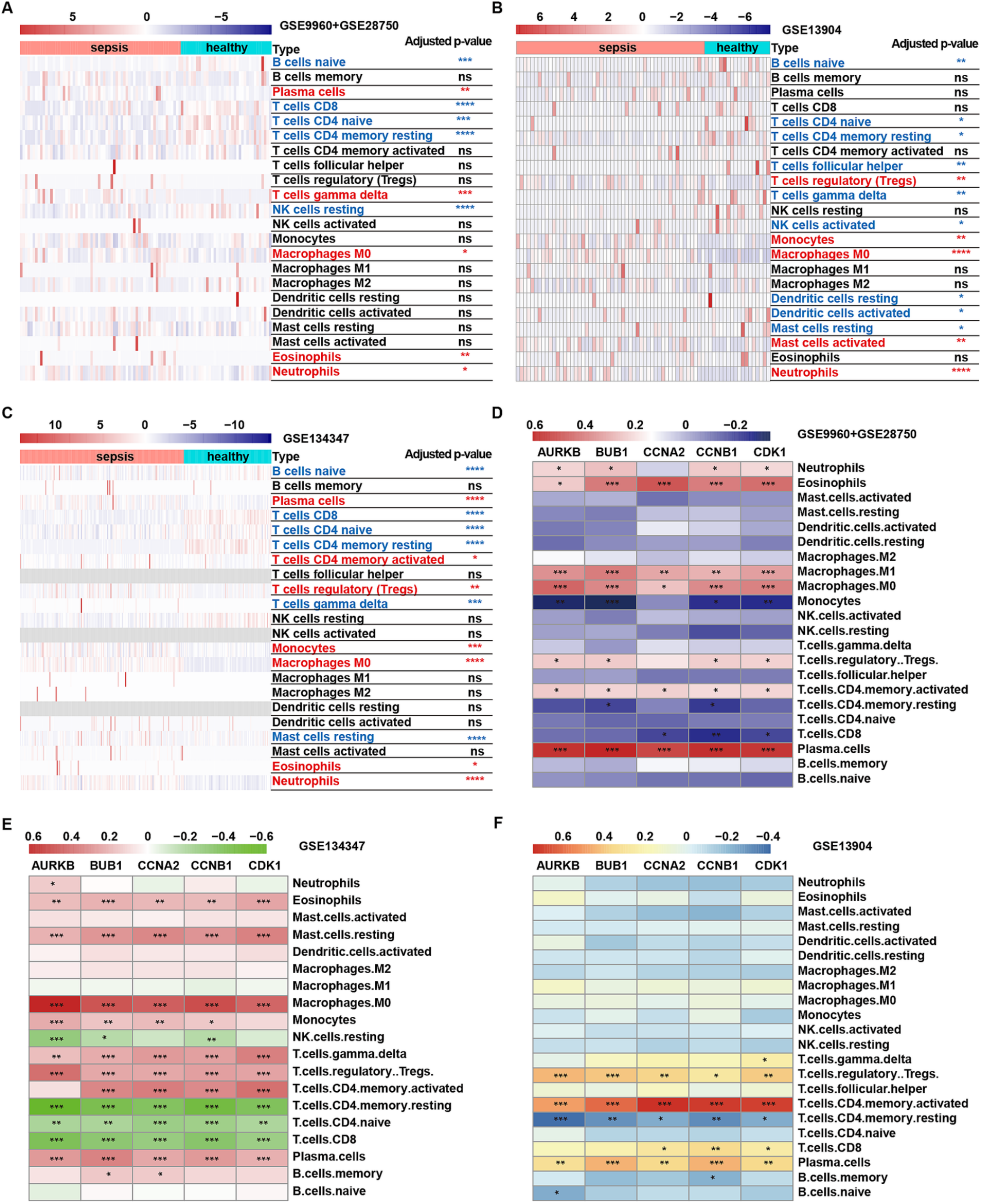
*CDK1*, *CCNB1*, *CCNA2*, *AURKB*, *BUB1* were correlated with immune cell infiltration in sepsis. **(A–C)** The differences of 22 immune cell infiltration between sepsis and healthy samples in the training set **(A)**, GSE13904 **(B)**, and GSE134347 **(C)** cohorts. Red fonts indicate immune cell types that were significantly increased in sepsis compared to healthy controls, while blue fonts indicate those significantly decreased. **(D–F)** The correlation of 5 genes with 22 immune cell infiltration in the training set **(D)**, GSE134347 **(E)**, and GSE13904 **(F)** cohorts. * means *p* < 0.05, ** means *p* < 0.01, *** means *p* < 0.001, **** means *p* < 0.0001. Significance markers represent adjusted p values (Benjamini-Hochberg correction).

Sepsis is a systemic inflammation caused by deregulation of the host immune response during infection. Thus, to investigate the relationship between these genes and the immune cell infiltration in sepsis, we calculated the different immune cell infiltration of samples in the different cohorts using the CIBERSORT algorithm. After CIBERSORT filtering, all samples from the training set and validation cohorts (GSE13904, GSE134347) met the quality threshold (*p* < 0.05) and were included in the analysis. In the training set, the proportions of plasma cells, gamma-delta T cells, M0 macrophages, eosinophils, and neutrophils were significantly increased and the proportion of naive B cells, CD8 + T cells, naive CD4 + T cells, resting memory CD4 + T cells, and resting natural killer (NK) cells were observably decreased in the sepsis group compared to the healthy group (Figure 8A, *p* < 0.05). In addition, the proportions of M0 macrophages and neutrophils were also increased, while the proportions of naive B cells, CD8 + T cells, naive CD4 + T cells, resting memory CD4 + T cells, and resting mast cells were reduced in the sepsis group compared to the healthy group in the GSE13904 and GSE134347 cohorts (Figures 8B,C, *p* < 0.05).

The Pearson correlation analysis showed that the proportions of M0 macrophages and plasma cells were significantly positively correlated with the expression *CDK1*, *CCNB1*, *CCNA2*, *AURKB*, and *BUB1* in the training set and GSE134347 cohort (Figures 8D,E, Benjamini-Hochberg, adjusted *p* < 0.05). The proportion of CD8 + T cells was significantly negatively correlated with *CCNA2*, *CCNB*1 and *CDK1* expression, and the proportion of resting memory CD4 + T cells showed a negative correlation with *BUB1* and *CCNB1* expressions in the training set, GSE134347 and GSE13904 cohort (Figures 8D–F, Benjamini-Hochberg, adjusted *p* < 0.05). However, given that the five genes are cell-cycle related, these correlations may partly reflect changes in cellular composition rather than direct immune regulatory relationships.

## 4 Discussion

Sepsis is defined as life-threatening organ dysfunction resulting from a dysregulated host response to infection (31). In this study, we identified five genes (*CDK1*, *CCNB1*, *CCNA2*, *AURKB*, and *BUB1*) closely associated with sepsis, which also showed correlations with immune cell infiltration.

Firstly, we screened a total of 1,463 genes associated with sepsis pathogenesis by gene network. Among these, 5 genes (*CDK1*, *CCNB1*, *CCNA2*, *AURKB*, and *BUB1*) were identified as candidate genes associated with sepsis. CDK1, a member of the cyclin-dependent kinase family, serves as a master regulator of the cell cycle and binds classical cyclins to coordinate mitotic progression (32). Cyclin B1 (CCNB1) and Cyclin A2 (*CCNA2*) are members of the cyclin protein family, and they play key roles in cell cycle regulation. In mitotic cell cycles, *CCNB1* is expressed beginning in late S phase, and increased in G2 phase and peaked at mitosis, and it plays a critical role in G2/M phase transition during the cell cycle (33). *CCNB1* could bind to the cyclin-dependent kinase. *CCNB1* binds to *CDK1* to form the *CCNB1*/*CDK1* complex, which stays in the nucleus under p21 induction, causing cell cycle arrest (34). *CCNA2* is expressed in all proliferating cells. In progress of cell cycle, *CCNA2* begins to be expressed in the S phase, before it binds and activates *CDK1* and *CDK2*, thereby driving S phase progression (35), and it is rapidly degraded upon entry of cells into mitosis (36). In addition, Almansa et al. have demonstrated that the expression levels of *CDK1*, *CCNB1*, and *CCNA2* were directly correlated with the extent of organ failure and mortality of sepsis (37). In the present study, we observed that *CDK1*, *CCNB1*, and *CCNA2* showed potential diagnostic value in some datasets, though these findings are correlational and require further validation. In addition, the *CDK1* and *CDK2* proteins were expressed in human neutrophils (38). Hence, it is reasonable to observe the *CDK1* expression was not correlated with infiltration of neutrophil in our study.

Aurora kinases (AURKA, AURKB, AURKC) and budding uninhibited by benzimidazole 1 (BUB1) are the mitotic Serine/Threonine kinases (39, 40). Aurora kinase is firstly identified in Drosophila. The main function of Aurora kinase is regulation the cellular mitosis (41). AURKA mainly controls the maturation of the centrosome and assembly of bipolar spindle (42), AURKB is a member of chromosomal passenger complex (CPC), and its functions contain regulation the interactions of chromosome with microtubules, cytokinesis and maintenance the stability of spindle (43). BUB1 is initially found in budding yeast (44). As a conserved kinase, *BUB1* is essential for the spindle assembly checkpoint (40). In addition, BUB1 could facilitate the alignment of chromosome (45). Noteworthily, the AURKB and BUB1 cooperate to maintain the integrity of BubR1-L (46). Like *CDK1*, *CCNB1*, and *CCNA2*, most of the current researched revealed the role of *AURKB* and *BUB1* in tumor (47–50). Interestingly, the BUB1, AURKA and AURKB were co-expressed in ovarian serous carcinoma at advanced-stage (51). Matsubara et al. have found that the BUB1 expression was correlated with phosphorylation of Akt and JNK, and *BUB1* insufficiency could prevent the acute renal failure in sepsis by regulating the thrombomodulin and tissue factor expression (52). However, the role of *AURKB* in sepsis has rarely been reported. In present study, we found that the *AURKB* and *BUB1* exhibited diagnostic value in sepsis. Given the established roles of these five genes in mitosis, it is possible that they may influence the progression of sepsis through cell-cycle-related pathways. However, this hypothesis requires further investigation using functional experiments, such as gene knockdown or overexpression in relevant sepsis models.

It is worth noting that all five hub genes identified in this study are canonical cell-cycle or mitosis-related genes. This raises the possibility that their upregulation in sepsis may partly reflect changes in blood cell composition or proliferative signals rather than sepsis-specific pathophysiological mechanisms. Indeed, our immune infiltration analysis revealed significant positive correlations between these genes and M0 macrophages, as well as negative correlations with CD8 + T cells and resting memory CD4 + T cells. These correlations suggest that the observed expression patterns of *CDK1*, *CCNB1*, *CCNA2*, *AURKB*, and *BUB1* may be influenced by shifts in immune cell subsets during sepsis. Therefore, while these genes show diagnostic potential, their specificity to sepsis biology remains to be further investigated in cell-type-resolved analyses. An additional methodological consideration arises from our multivariable logistic regression model. Although *CCNB1* was consistently upregulated in sepsis samples across all datasets, it exhibited a negative regression coefficient in the five-gene model. Our VIF analysis confirmed that this was not attributable to multicollinearity, as all VIF values were below the conventional threshold of 5. This observation illustrates an important principle in multivariable modeling: the coefficient of a predictor represents its unique, partial contribution to the outcome while holding all other predictors constant. Thus, after accounting for the expression levels of *CDK1*, *CCNA2*, *AURKB*, and *BUB1*, the relative level of *CCNB1* contributes negatively to the predicted probability of sepsis. This discrepancy between univariate and multivariable effects is not uncommon when predictors are correlated, even in the absence of problematic multicollinearity. It underscores the importance of interpreting multivariable models holistically rather than focusing on the direction of individual coefficients. The model as a whole learns a complex decision boundary that achieves improved discriminative performance compared to single-gene classifiers.

Sepsis induces immune dysregulation of the innate and adaptive immune systems, which triggers persistent pro-inflammatory and anti-inflammatory pathways that ultimately lead to tissue and organ dysfunction (53). In our study, we found that M0 macrophages, neutrophils, naive B cells, CD8 + T cells, and resting memory CD4 + T cells were concurrently dysregulated in sepsis, these observations align with previous studies (54). In sepsis, an increase in the proportion of M0 macrophages has been observed, whereas the proportions of naïve CD4 + T cells, CD8 + T cells and resting memory CD4 + T cells have been shown to decrease (55). Macrophages are capable of producing a variety of inflammatory cytokines, including TNF-*α*, IL-1, and IL-6 (56). However, the pro-inflammatory cytokines produced by macrophages can be detrimental during sepsis (57). The expression of MHCII proteins on macrophages’ surfaces was diminished in patients with sepsis, leading to ineffective T cell activation and worsening of the sepsis condition (58). The proportions of CD8 + T cells, and resting memory CD4 + T cells were reduced, which may be related to the massive apoptosis of lymphocytes during sepsis (59). In the early stages of sepsis, lymphocytes undergo apoptosis, resulting in immunosuppression and lymphopenia. In the early stages of sepsis, lymphocyte apoptosis leads to lymphopenia and immunosuppression (60). Moreover, we found that M0 macrophages proportions were positively correlated with the expression *CDK1, CCNB1, CCNA2, AURKB, BUB1*, and *CD8* + T cell proportions were negatively correlated with *CCNA2*, *CCNB1* and *CDK1* expression, and the proportion of resting memory CD4 + T cells had negative correlation with *BUB1* and *CCNB1* expressions. Given the crucial role of immune cells in the development of sepsis, the observed correlations between the expression of these five genes and immune cell proportions suggest a potential link between these genes and immune regulation. However, whether these genes directly regulate immune cell functions or simply reflect changes in cell composition remains to be determined through functional studies.

Several limitations of this study should be acknowledged. First, the scRNA-seq analysis included only three adult and three pediatric sepsis samples without healthy controls, limiting the generalizability of the observed age-related differences. Second, the retrospective nature of the data may introduce bias, and prospective studies are warranted. Third, the immune infiltration analysis provides estimated rather than directly measured immune cell abundances, and the observed correlations may partly reflect changes in blood cell composition rather than sepsis-specific mechanisms. Additionally, given that the five hub genes are cell-cycle related, their correlations with immune cell proportions should be interpreted cautiously, as these associations may be driven by differences in cellular proliferation rates or cell composition rather than specific immune regulatory functions. Fourth, the healthy control group in the qRT-PCR validation was not age- or sex-matched to the sepsis group, which may have introduced bias. Age- and sex-matched controls are recommended in future validation studies.

## 5 Conclusion

In this study, we identified five sepsis-associated genes (*CDK1*, *CCNB1*, *CCNA2*, *AURKB*, and *BUB1*). A logistic regression model based on these five genes showed preliminary discriminative potential for sepsis. Exploratory scRNA-seq analysis showed broader cell-type distribution in pediatric samples (B cells, monocytes, and T cells) compared to adults (largely restricted to T cells). These findings suggest that these five genes are candidate biomarkers for sepsis, but as the results are correlational, they should be interpreted as hypothesis-generating and warrant further validation in larger cohorts and functional studies.

## Data Availability

The original contributions presented in the study are included in the article/Supplementary material, further inquiries can be directed to the corresponding authors.

## Footnotes

1
https://www.ncbi.nlm.nih.gov/geo/

2
https://www.gsea-msigdb.org/gsea/msigdb

## Supplementary material

SUPPLEMENTARY FIGURE S1 Merging GSE9960 and GSE28750 datasets and removing batch effect. **(A)** The boxplot of the normalized data. **(B)** PCA results before batch removal for multiple datasets. Different colors represent different datasets. **(C)** PCA results after batch removal.

SUPPLEMENTARY FIGURE S2 The result of soft threshold β in WGCNA.

SUPPLEMENTARY FIGURE S3 A protein-protein interaction (PPI) network.

SUPPLEMENTARY FIGURE S4 Logistic regression model incorporating the five candidate genes for sepsis diagnosis. **(A)** Component-plus-residual plots for each of the five genes (*CDK1*, *CCNB1*, *CCNA2*, *AURKB*, *BUB1*) in the logistic regression model. **(B)** Calibration curve of the five-gene logistic regression model in the training set. **(C,D)** ROC curves of the five-gene logistic regression model in the training set (GSE9960+GSE28750) **(C)** and validation cohort (GSE65682) **(D)**. **(E,F)** ROC curves of the five-gene logistic regression model in the GSE154918 **(E)** and GSE131411 datasets **(F)**.

SUPPLEMENTARY TABLE S1 Sepsis-associated genes identified in the yellow, black, and blue modules by WGCNA.

SUPPLEMENTARY TABLE S2 The results of GSVA and GSEA.

SUPPLEMENTARY TABLE S3 The results of top 10 genes in PPI network.
