# Supplementary figures and images for: Identification and validation of novel candidate genes with diagnostic value for sepsis via weighted gene co-expression network analysis
Source: Front Med (Lausanne). 2026 Jun 29;13:1817161. doi: 10.3389/fmed.2026.1817161 (PMC13357434; doi:10.3389/fmed.2026.1817161)

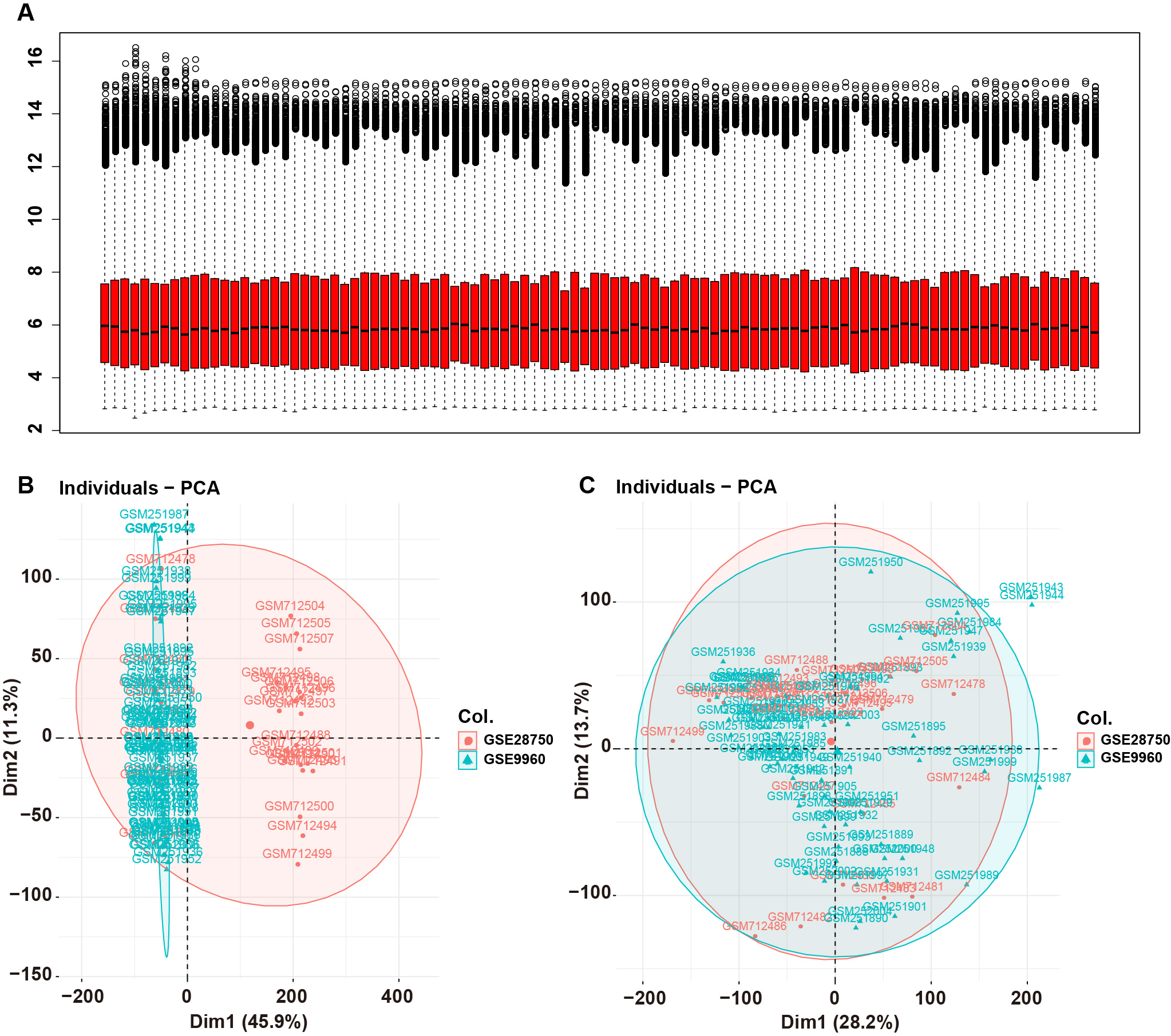

Supplement: SUPPLEMENTARY FIGURE S1 — Merging GSE9960 and GSE28750 datasets and removing batch effect. (A) The boxplot of the normalized data. (B) PCA results before batch removal for multiple datasets. Different colors represent different datasets. (C) PCA results after batch removal. [file Image_1.jpeg]

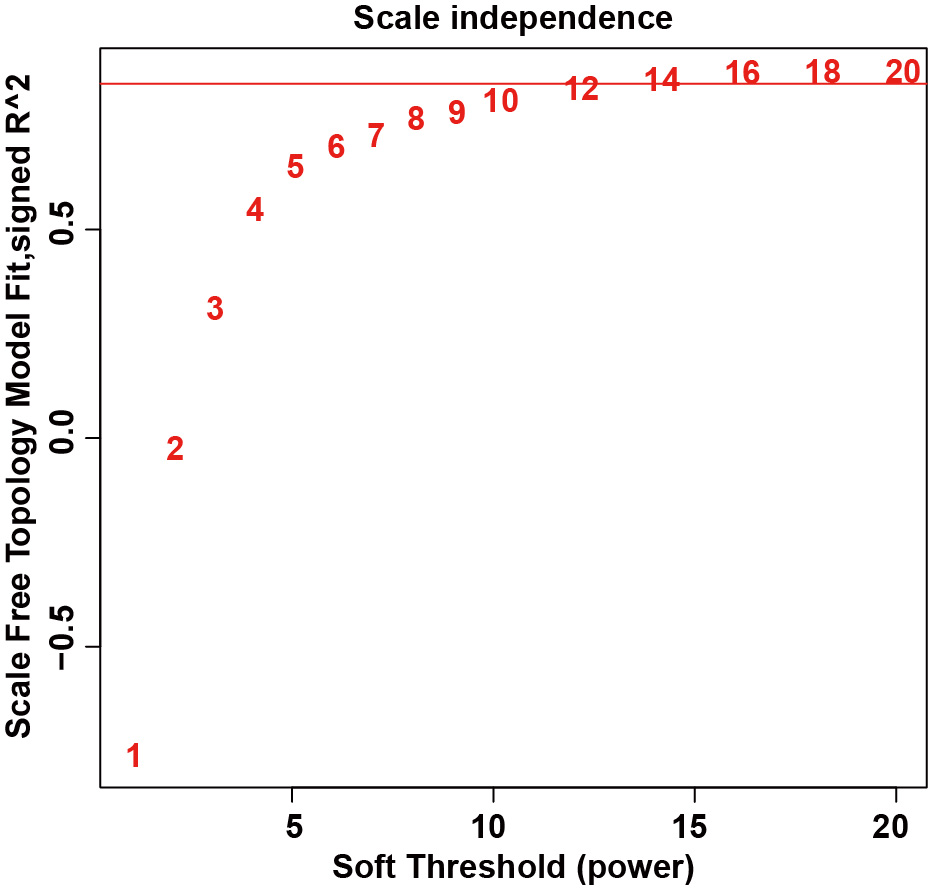

Supplement: SUPPLEMENTARY FIGURE S2 — The result of soft threshold β in WGCNA. [file Image_2.jpeg]

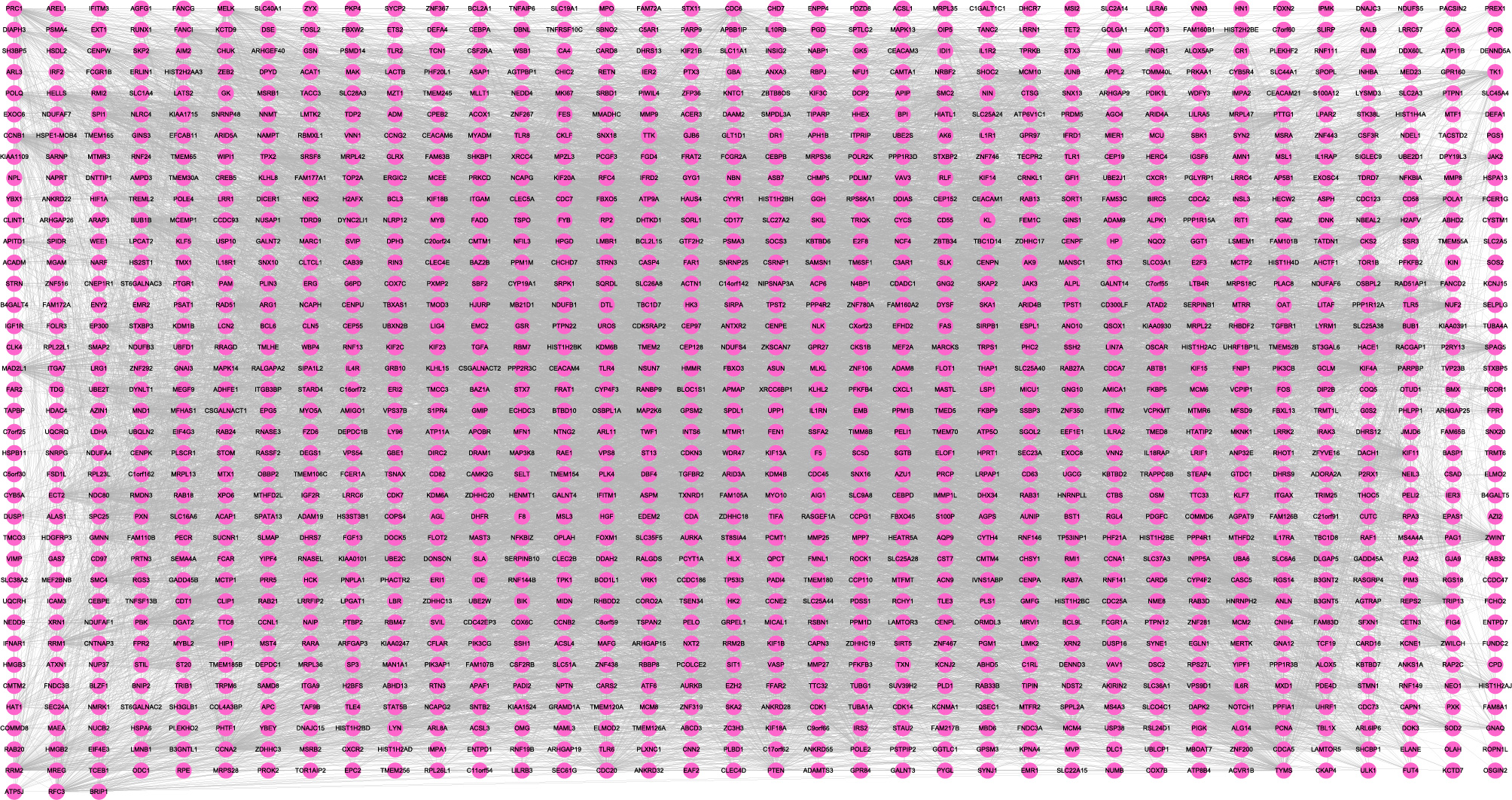

Supplement: SUPPLEMENTARY FIGURE S3 — A protein-protein interaction (PPI) network. [file Image_3.jpeg]

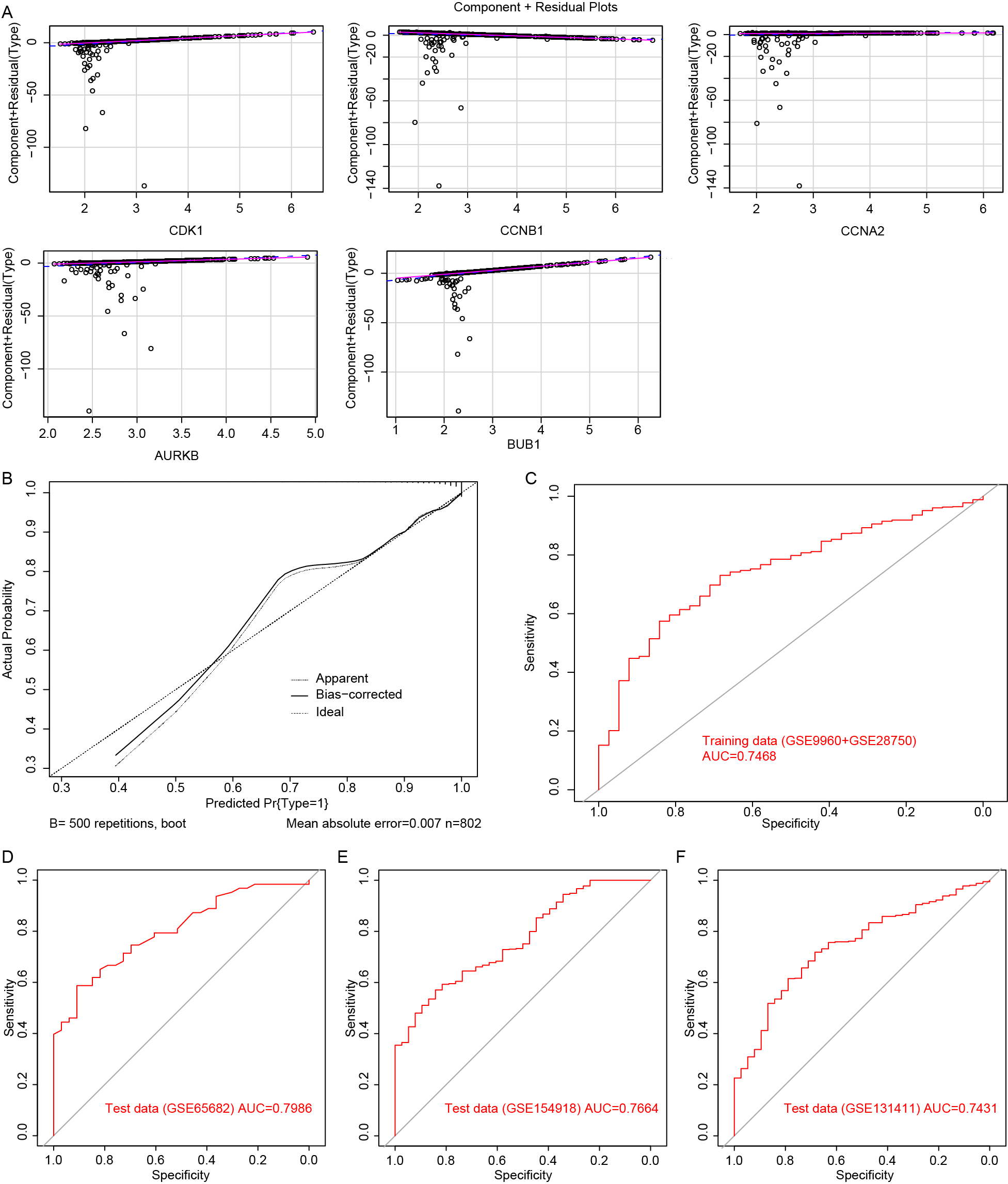

Supplement: SUPPLEMENTARY FIGURE S4 — Logistic regression model incorporating the five candidate genes for sepsis diagnosis. (A) Component-plus-residual plots for each of the five genes (CDK1, CCNB1, CCNA2, AURKB, BUB1) in the logistic regression model. (B) Calibration curve of the five-gene logistic regression model in the training set. (C,D) ROC curves of the five-gene logistic regression model in the training set (GSE9960+GSE28750) (C) and validation cohort (GSE65682) (D). (E,F) ROC curves of the five-gene logistic regression model in the GSE154918 (E) and GSE131411 datasets (F). [file Image_4.tif]
